# Compassion fatigue and associated factors among nurses working in Jimma Zone public hospitals, southwest Ethiopia: A facility based cross-sectional study

**DOI:** 10.1371/journal.pone.0312400

**Published:** 2025-01-16

**Authors:** Duguma Debela Ganeti, Bikila Dereje Fufa, Ebissa Bayana Kebede, Sheka Shemsi Seid, Birhanu Wogane Ilala, Nuritu Bacha Benti, Yeshitila Belay Belachew

**Affiliations:** 1 School of Nursing, College of Health Sciences, Assosa University, Assosa, Ethiopia; 2 School of Nursing, Faculty of Health Sciences, Jimma University, Jimma, Ethiopia; 3 School of Midwifery, College of Health Science, Wollege University, Nekemte, Ethiopia; University of Auckland, NEW ZEALAND

## Abstract

**Background:**

Nurses are at risk of developing compassion fatigue, which has negative impacts on their well-being, quality care and leads to patient mortality and a financial burden on the healthcare system. However, data on compassion fatigue is scarce in Africa, particularly Ethiopia. Therefore, this study aimed to assess level compassion fatigue and associated factors among nurses in Jimma Zone public hospitals, Ethiopia.

**Method:**

A facility-based cross-sectional study was employed from May 25 to June 25, 2023. A systematic sampling technique was employed to select among 422 respondents. Data were collected using pretested self-administered questionnaires. Professional Quality of Life Scale-5 was used for measuring compassion fatigues. Data were entered using Epi data version 4.6 and analyzed using SPSS version 25. Linear regression were done to identify factors associated with compassion fatigue. Statistically significant was declared at a p-value of ≤ 0.05 with 95% CI.

**Result:**

From a total of 422 respondents, 412(97.6%) of them gave complete responses. 47% of respondents, had a moderate level of compassion fatigue. Total experience [β = -0.04; 95%CI (-0.06, -0.01); p = 0.005], perceived social support [β = -0.13; 95% CI (-0.17, -0.08); p<0.001], self-compassion [β = -0.09; 95% CI (-0.14, -0.03); p = 0.003], support seeking [β = -0.23; 95% CI (-0.42, -0.04 p = 0.017], emergency ward [β = 0.36; 95% CI (0.2, 0.51); p <0.001], ICU [β = 0.38; 95% CI (0.21, 0.54); p<0.001], pediatric ward [β = 0.23; 95% CI (0.10, 0.36); p < 0.001] and average sleep hours per day [β = 0.46; 95% CI (0.35, 0.57); p<0.001] were statistically signifantly factors.

**Conclusion and recommendation:**

The study revealed that one in four nurses had high level of compassion fatigue. The factors associated were work experience, perceived social support, self-compassion, coping strategies, work unit, and sleep hours. Therefore, stakeholders including hospital managers should implement targeted strategies to prevent compassion fatigue including training on coping strategy and, self-compassion and creating culture of team work among nurses.

## Introduction

Nurses contribute to national and global health by providing patient care, participating in rehabilitation, supporting patients and their families, and advocating health education [1, 2]. This broad and multifaceted work requires direct, prolonged exposure to the suffering of the patients and puts them under considerable occupational hazards such as compassion fatigue (CF) [1, 3]. CF was first described in health care professionals as a unique form of burnout by Joinson in 1992 to describe an occupational hazard specific to caring professionals such as nurses, counselors, ministers, and other caregivers [3]. Nurses are at a higher risk of CF among healthcare professionals (HCPs) as their profession requires continuous and repeated exposure to patients’ suffering, high-stress environments, and the constant giving of self, constancy, and proximity to tragedy over time and inability to remove themselves from their source of distress [3, 4]. CF is also called secondary traumatic stress (STS), which results from exposure to the trauma experienced by patients rather than to the trauma itself [5].

Compassion fatigue is a preventable state of physical, emotional, and spiritual exhaustion induced by witnessing and absorbing the death and suffering of other peoples without establishing boundaries and self-care practices [4, 6]. Compassion fatigue leads to multiple symptoms, and these can be categorized into seven domains: cognitive (e.g., decreased concentration, disorientation, apathy), emotional (e.g., powerlessness, anxiety, numbness), behavioral (e.g., irritability, withdrawal, hypervigilance), spiritual (e.g., loss of purpose, questioning prior beliefs, lack of self-satisfaction), personal relations (e.g., decreased interest in intimacy, isolation from others, increased interpersonal conflicts), somatic (e.g. sweating, rapid heartbeat, dizziness) and lastly work performance (e.g. lowered motivation, absenteeism, exhaustion) [7, 8].

CF negatively affects nurses’ physical and mental well-being, patients and health care system. It also leads to maladaptive behaviors such as substance use, strained personal relationships, absenteeism, attrition, avoidance of patients, impaired clinical judgment and compromise patient care [4, 9, 10]. Additionally, it can cause mental health problems, like depression, which can result in suicidal death and adversely affect the nurses’ private life, their family and patients around them [11–13].

Compassion fatigue undermines nurses’ compassion and empathy and affects quality of nursing care, resulting in poorer delivery of health services and increased patient mortality rate, job dissatisfaction, high use of sick leave, turnover and contributes to the global shortage of nurses, which puts financial burdens on the healthcare system [9–11, 14–16].

Compassion fatigue is progressively becoming a problem in the global healthcare system, with evidence from a systematic review revealing that the prevalence of CF among nurses increased from 2010 to 2019 [17]. Another systematic review conducted across six various countries (China, United States, Spain, Portugal, Korea, and Canada) revealed that almost one-fourth of nurses experienced high levels of CF, and the severity of CF differed across geographical regions, with nurses in Asia having higher CF compared to America [18]. The prevalence of CF become more severe among nurses working in low and middle-income countries, with a study from Uganda reporting that half of the nurses experienced high levels of CF. Although the level of CF among nurses in Ethiopia is not well unknown, nurses in Ethiopia deals with a disproportionate nurse-patient ratio, unfavorable work environments, and management issues, which might contribute to the development of CF [19, 20].

Studies have revealed that the levels of CF experienced by nurses varied based on their socio-demographic characteristics, work environment, social factors and personal factors [6, 9, 21–23]. Compassion fatigue can happen to any nurse, at any time during the job course, but some nurses at higher risk of developing CF [24]. A systematic review reported that the ICU nurses suffered from severe compassion fatigue [17]. Another study from Portugal showed 58.6% of nurses from an emergency and urgent care unit in had high levels of CF [25]. Study from Uganda, showed that 49.11% of nurses working in frontline during Covid-19 had high levels of CF [26].

Despite these negative consequences on nurses’ well-being, patients, and healthcare organizations, nurses there are limited studies on the level and related factors of CF among nurses in our country and Africa as well. Existing studies in Africa on nurses’ CF levels concentrated on nurses serving in specific units [26, 27]. Moreover, variables related to the work environment, such as a healthy work environment (HWE) and personal factors like coping strategy and self-compassion were not adequately explored. Therefore, this study aimed at assessing the level of CF and associated factors among nurses working in public hospitals across multiple in patient units by including less studied variables in the Jimma Zone.

## Materials and methods

The study was conducted in all public hospitals found in the Jimma Zone, one of the zones of the Oromia Regional State in southwest Ethiopia. Jimma City serves as the capital city of the Jimma Zone and covers an area of 15,568.58 square kilometers, situated 352 kilometers to the southwest of Addis Ababa. The zone comprises 21 woredas and two administration towns, Jimma City and Agaro Town, with 555 Kebeles, of which 515 are rural and 40 are urban Kebeles [28].

The zone has nine public hospitals, 122 health centers, and 512 health posts. Out of the nine public hospitals, one is a tertiary hospital, three are general hospitals, and five are primary hospitals. These hospitals are Jimma Medical Center (JMC), Shenan Gibe General Hospital, Agaro General Hospital, Limu General Hospital, Nada Primary Hospital, Seka Chekorsa Primary Hospital, Setema Primary Hospital, Dimtu Primary Hospital, and Dedo Primary Hospital [28, 29]. Currently in the hospitals there total of 856 nurses working in inpatient units of Jimma Zone public hospitals. The public hospitals in Jimma Zone provide services to more than 2,488,155 population [28]. JMC is the only tertiary hospitals in southwest Ethiopia and it provides specialized clinical services to in-patients and out-patients on a referral system in the southwest The study was conducted from May 25 to June 25, 2023.

### Study design and population

A facility-based cross-sectional study design was employed. Nurses who had at least six months of experience working in inpatient and emergency wards of hospitals. Nurses who were on leave (maternal, annual, and sick leave) and not in active clinical care roles were excluded from the study.

### Sample size determination and sampling procedures

The sample size was determined using a single population proportion formula with the following assumptions: Z = the standard average deviation at 95% confidence level; = 1.96, d = margin of error that can be tolerated, 5% (0.05), and P is assumed to be 0.5 (50%) because there is no previous similar study on CF in our country.


$$\text{n}=\frac{\left(Z\frac{\alpha }{2}\right)2\;\text{p}\;\left(1-\text{p}\right)}{\text{d}\;2}=\frac{\left(1.96\right)2\;0.5\left(1-0.5\right)}{\left(0.05\right)2}=384.$$

The calculated sample size was 384. By adding a 10% non-response rate, the final sample size was 422.

A systematic sampling technique was employed to pick nurses from each hospital after samples were proportionally allocated for all public hospitals. The list of eligible nurses working in the selected unit of all public hospitals, which is 856, was taken and served as a sampling frame. Then, respondents were chosen every two intervals from the sampling frame (K = N/n = 856/422 = 2). The first respondent was selected using lottery methods.

### Study variables

#### Dependent variable

Compassion fatigue.

#### Independent variables

Socio-demographic characteristics: Age, sex, marital status, work experience, educational status, monthly income, number of children and work experience.

Working related factors: HWE, work unit, and hours worked per week.

Social factors: Perceived social support.

Personal related factors: Self-compassion, coping strategy, job satisfaction, and sleep hours.

### Data collection tool

Data was collected by a structured self-administered questionnaire abstracted by reviewing different peer-reviewed studies [21, 30–34].

The tool was prepared and administered in the English language. The questionnaire consists of seven parts. The first part includes questions on the sociodemographic and work-related details of the study respondents.

The second part consist questions about MSPSS. MSPSS scale is the self-report measurement of perceived social support (emotional, instrumental, informational, and appraisal) from three sources of individuals’ social lives: family, friends, and significant others. The scale contains 12 items with a 7-point Likert-type scale for its measurements, with ratings from "1 = very strongly disagree" to "7 = very strongly agree. It measures the perceived level of support from family, friends, and a significant other) [33].

Part three of questionnaire consists of the SCS-SF, which measures self-compassion on a five-point Likert-type scale, with response options ranging from 1 (almost never) to 5 (almost always). SCS-SF measures how people typically act towards themselves during times of great difficulty. It includes self-kindness, self-judgment, mindfulness, common humanity, isolation, and over-identification. Subscale scores are computed by adding item scores. A total self-compassion score is computed by reversing the negative subscale items (self-judgment, isolation, and over-identified), and a grand mean of all six subscales was calculated [30].

The fourth part consists of questions for measuring nurses’ ratings of HWE. The questionnaire was the 18-item AACN HWE nurses’ ratings of the 6 HWE components (3 questions per component). Those components are skilled communication, true collaboration, effective decision-making, appropriate staffing, meaningful recognition, and authentic leadership. All items used a 5-point Likert-type scale, with response options ranging from 1 (strongly disagree) to 5 (strongly agree) [31].

The fifth part of the study involved a questionnaire for measuring job satisfaction. It is measured by the MSQ short form, which consists of twenty items with a 5-point Likert-type scale for responses. The scale ranges from 1, indicating strong dissatisfaction, to 5, indicating strong satisfaction [34].

The sixth part consists of questions about the coping strategy of nurses. It is measured by the CSI, developed by Amirkhan, and includes three subcategories: seeking social support, problem-solving, and avoidance. Each subcategory consists of 11 items, with responses ranging from "not at all" to "a little" to "a lot" on subscales of 1–3 [21].

The seventh part of the questionnaire was used to measure CF. It was measured using the ProQOL-5, anchored by a five-point Likert scale. This instrument was chosen because of its ability to measure CF as individual concepts, good internal validity, and reliability across studies in the last 30 days [32].

### Data collection procedure

An anonymous self-administered structured questionnaire was used to collect data from study respondents. Data collection was facilitated by four BSc nurses who have previous data collection experience, and one MSc nurse was recruited as a supervisor. The data facilitator distributed the questionnaires to the respondents in their free time, and respondents were encouraged to read and respond carefully during their free time to ensure that all items were thoroughly completed. Lastly, the data facilitator collected the filled data after checking for the completeness of the data.

### Operational definition and definition of terms

#### Compassion fatigue

CF was measured by professional quality of life—5 (ProQOL 5). It contains ten items with five Likert scales and is categorized based on the sum score of the scale. CF raw score was transformed into standardized t-scores and categorized using the cut scores for the ProQoL-5 as -low, moderate, and high CF if the t-score is ≤42, 43–56, and ≥57, respectively [35].

#### Perceived social support

It is a measure of perception of social support (emotional, instrumental, informational, and appraisal) from family, friends, and significant others. It is measured by a multidimensional scale of perceived social support (MSPSS), which contains 12 items with seven Likert scale and is categorized based on the mean of scale, with a score ranging from 1 to 2.99 considered low support; a score of 3 to 5 as moderate support, and a score from 5.1 to 7 considered high support [33].

#### Healthy work environment

It is measured by the 18-item American Association of Critical-Care Nurses (AACN) HWE of nurses’ ratings. AACN provides cutoff points for assessment based on overall mean scores (1–2.99—Needs Improvement, 3–3.99 –Good, 4–5 –Excellent) [36].

#### Self-compassion

Self-compassion is an emotionally positive self-attitude that protects against the negative consequences of self-judgment, isolation, and rumination. It measures how people typically act toward themselves during times of great difficulty [37]. It is measured by the Self-Compassion Scale Short Form (SCS-SF). It is measured by a 1–5 Likert scale and categorized as a score of <2.5 indicates low self-compassion, 2.5–3.5 indicates moderate, and >3.5 indicates high self-compassion [30].

#### Job satisfaction

Measured by the Minnesota Satisfaction Questionnaire (MSQ) short form and categorized based on sum score. Respondents who scored more than 60 on the sum of all the satisfaction scale items were considered satisfied with their jobs. Those who scored 60 and below were taken as dissatisfied [34].

#### Coping strategies

Coping strategy is a deliberate and effortful attempt to manage stress. The coping strategy indicator (CSI) measured nurses’ coping strategies. It included three subcategories: seeking social support, problem-solving, and avoidance. Higher scores for a coping strategy indicate greater use of the coping strategy [38].

Average sleep hours: was obtained by asking for average sleep hours per day, categorized as (≤ 6 hours or > 6 hours) based on recommended daily sleep hours [39].

### Data quality assurance

Data quality was assured by providing a one-day orientation for data facilitators and supervisor and pretesting the tool before data collection. Orientation covered the techniques of data collection, the purpose of data collection, the content of the questionnaires, how to approach the respondents, and how to address any difficulties that may arise during data collection. Throughout the data collection period, the principal investigator and supervisor provided daily supervision to ensure the data’s completeness and quality. Upon completion of the data collection, each questionnaire was thoroughly reviewed to check for completeness, and data cleaning was done by checking for inconsistencies, numerical errors, and missing parameters.

A pretest was done at Bedele Hospital one week before the data collection to ensure the consistency and language clarity of the tool used. The pretest was conducted by taking 5% (21 participants) of the total sample size and administering the questionnaire to them. Following the pretest, the language clarity of the tool was evaluated, and necessary changes were made. Furthermore, the inter-item consistency of the tool was assessed using a Cronbach’s coefficient alpha test, yielding a coefficient of 0.88 for CF, 0.84 for perceived social support, 0.71 for self-compassion, 0.93 for HWE, 0.92 for CSI and 0.89 for MSQ.

### Data processing and analysis

After the data collection, data were rechecked for completeness, cleaned, coded, and entered into Epidata version 4.6, and then exported to SPSS version 25 for analysis. Appropriate coding was done at each step for the variables. A descriptive analysis was used to present the factors of sociodemographic, personal, social, and work-related and the prevalence of CF (frequencies and percentages for categorical variables and with mean ± SD for continuous variables). Dummy variables were created for categorical variables with two and more than two categories.

Compassion fatigue scores were generated by transforming raw data into standardized t-scores and categorized using the cut scores for the ProQOL-5 manual as low, moderate, and high CF if the t-score is ≤ 42, 43–56, and ≥ 57, respectively [32]. However, because of the nature of variables, in inferential analysis, the mean scores of CF on the scale were used to increase the statistical power to detect associations among the CF and independent variables [40].

Multiple linear regression analysis was used to examine variables associated with CF. Basic assumptions of linear regression were checked before fitting the model. The data has fulfilled the basic assumptions of linear regression analysis. For normality tests, both Kolmogorov-Smirnova and Shapiro-Wilk had p-values of 0.156 and 0.116, respectively), indicating that the data was normally distributed. The linearity of the data was also confirmed with a scatter plot. independence of error was shown with a Durbin-Watson statistics p-value of 1.777 (within the acceptable range of 1.5–2.5). Additionally, the absence of outliers was confirmed, with no values falling above or below the plot bars on the box plot. Lastly, multi-collinearity was not an issue, as all values of VIF were below the cutoff point of 10.

First, simple linear regression was run at a 25% significance level to screen out potentially significant independent variables. Subsequently, a multiple linear regression model was run by including variables with a p-value of ≤ 0.25 from the simple linear regression. In the final model, p-value, unstandardized regression coefficients (ß), and 95% CI of ß were used to measure the presence and strength of association between CF and independent variables. Then, variables with p-value ≤ 0.05 were declared statistically significant associated factors with CF. Finally, the analysis result was presented in texts, tables, and graphs as appropriate.

### Ethics approval and consent to participate

Ethical clearance to conduct the study was obtained from the institutional review board (IRB) of the Institute of Health, Jimma University (Ref. No: JUH/IRB/401/23). The letter of support was also taken from JUH/IRB to each hospital, and permission to conduct the study was obtained from each hospital’s administration. The head nurse of each unit was informed about the purpose of the study, and then the head nurse informed the nursing staff. All respondents were informed of the study’s purpose, that the participation is entirely voluntary, and they also have the right to refuse participation. The study adhered to the Helsinki Declaration. Respondents were assured that their identifiers would not be linked to their responses and that the study was completely confidential. Additionally, written informed consent was obtained from all respondents before data collection.

## Result

### Socio-demographic characteristics of nurses

From the 422 sample size, 412 respondents were included, resulting in a response rate of 97.6%. Of the 412 respondents, the 240 (58.3%) were females. The average age of respondents was 32.43 ± 5.62 (SD) years, and 277 (67.20) of the respondents were below the age 35 years. Concerning marital status, 286 (69.4%) of the respondents were married.

Regarding education status, 350 (85%) nurses held a bachelor’s degree. Their average total experience and experience in the current unit were 6.76 ± 4.23 (SD) years and 2.1 ± 1.04(SD) years, respectively. The mean ± SD of working hours per week was 65.78 ± 9.707, ranging from 52 to 90 hours. On a number of children, 280 (68.0%), reported having at least one child.

Concerning working units, 64 (15.5%) participants worked in the pediatric ward. And on level of hospitals 218 (52.9%) of the respondents worked at referral hospitals. Regarding their monthly income, the mean ± SD salary of the respondents was 7099.33 ± 1438.00 ETB, with 286 (69.40%) receiving a monthly income ranging between 5000–8000 ETB birr (Table 1).

**Table 1:** Socio-demographic characteristics of nurses working in Jimma Zone public hospitals, southwest Ethiopia, 2023 (n = 412).

Variables	Category	Frequency	Percent (%)
Sex	Female	240	58.30
	Male	172	41.70
Age	<35	277	67.20
	≥35	135	32.80
Marital status	Married	286	69.40
	Single	100	24.30
	others@	26	6.30
Educational level	B.Sc.	350	85.00
	Diploma	54	13.10
	M.Sc.	8	1.90
Experience	1–5	196	47.60
	6–10	149	36.20
	>10	67	16.30
Monthly income	<5000	31	7.50
	5000–800	286	69.40
	>8000	96	23.30
Having children	No	132	32.00
	Yes	280	68.00
Working unit	Medical	50	12.1
	Surgical	60	14.6
	Pediatrics	64	15.5
	Emergency	48	11.7
	ICU	35	8.5
	Gynecology and obstetrics	29	7.0
	Neonatology	38	9.2
	OR	40	9.7
	Dental and maxillofacial	20	4.9
	Ophthalmology	28	6.8
Level of hospital	Referral	218	52.90
	General	88	21.40
	Primary	106	25.70

Others*: oncology = 2, dialysis = 3,edo/colonoscopy = 2, psychiatry = 2, @ = divorced and widowed. Msc = Masters degree, Bsc = Bachelors degree.

### Social factors

#### Perceived social support scale

The study revealed the overall mean ± SD score for perceived social support among the nurses was 3.83 ± 0.07, indicating that the mean score of perceived social support was moderate. From families’ perceived social support, 193 (46.8%) reported moderate levels of perceived family support. Of the total respondents, 151 (36.4%) nurses reported moderate perceived social support. Considering each subscale, the mean ± SD scores for the perceived family support (4.42 ± 1.52) were higher than those for perceived friends support (3.68 ± 1.51) and perceived significant others support (3.39 ± 1.62) (Table 2).

**Table 2:** Overall perceived social support and its subcomponents among nurses working in Jimma Zone public hospitals, southwest Ethiopia, 2023(n = 412).

Variables*	Mean ± SD	Scoring level		
		Low	Moderate	High
		N (%)	N (%)	N (%)
Overall perceived social support	3.83 ± 0.07	150(36.4)	151(36.7)	111(26.9)
Families’ perceived social support	4.42 ± 1.52	76(18.4)	193(46.8)	143(34.7)
Friends’ perceived social support	3.68 ± 1.51	159(38.6)	168(40.8)	85(20.6)
Significant others’ perceived social support	3.39 ± 1.62	202(49)	131(31.8)	79(19.2)

SD = Standard deviation.

* = Seven-point Likert scale measuring perceived social support.

### Personal-related factors

#### Self-compassion (SC)

**Fig 1:**
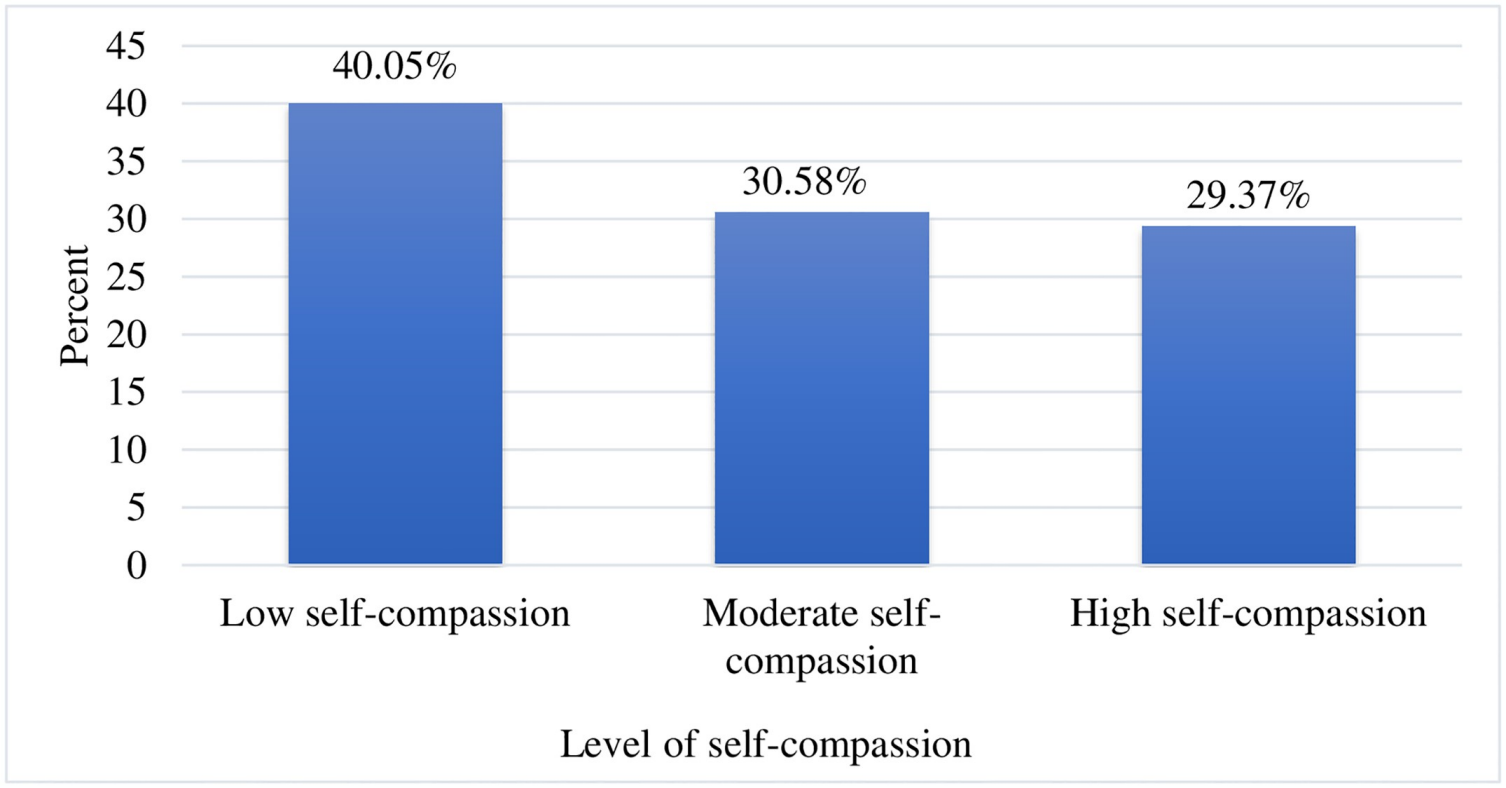
Level of self-compassion of nurses working in Jimma Zone public hospitals, southwest Ethiopia, 2023 (n = 412).

This study showed that 40.05% of nurses, had moderate to low levels of self-compassion (Fig 1).

#### Coping strategy indicator (CSI)

The result of the study shows that the mean scores for coping strategy indicators subscales were almost the same. However, the mean ± SD scores of problem-solving strategies (2.082 ± 0.57) were slightly higher than those seeking social support-oriented coping (2.075 ± 0.52) and avoidance (2.078 ± 0.36) coping strategies (Table 3).

**Table 3:** Descriptive statistics of coping strategy indicator items of nurses working in Jimma Zone public hospitals, southwest Ethiopia, 2023 (n = 412).

coping strategy indicator*	Mean ± SD
**Seeking Support**	***2*.*075(0*.*52)***
**Problem-Solving**	***2*.*082(0*.*57)***
**Avoidance**	***2*.*078(0*.*36)***

* = three-point Likert scale measuring coping strategy indicator.

#### Job satisfaction

**Fig 2:**
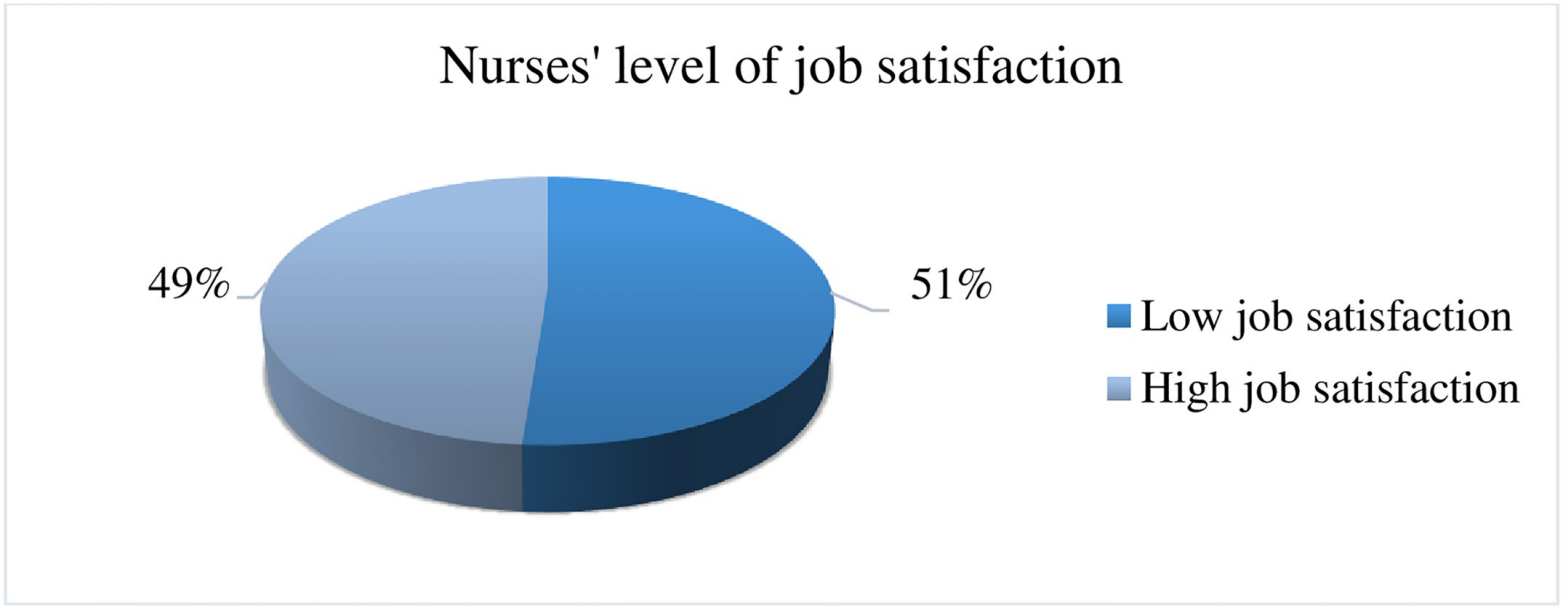
Job satisfaction of nurses working in Jimma Zone public hospitals, southwest Ethiopia, 2023 (n = 412).

The result of the study shows minimum and maximum scores were 29 and 95 respectively. More than half, 211(51%) of study nurses were dissatisfied with their jobs (Fig 2).

#### Sleep hours

**Fig 3:**
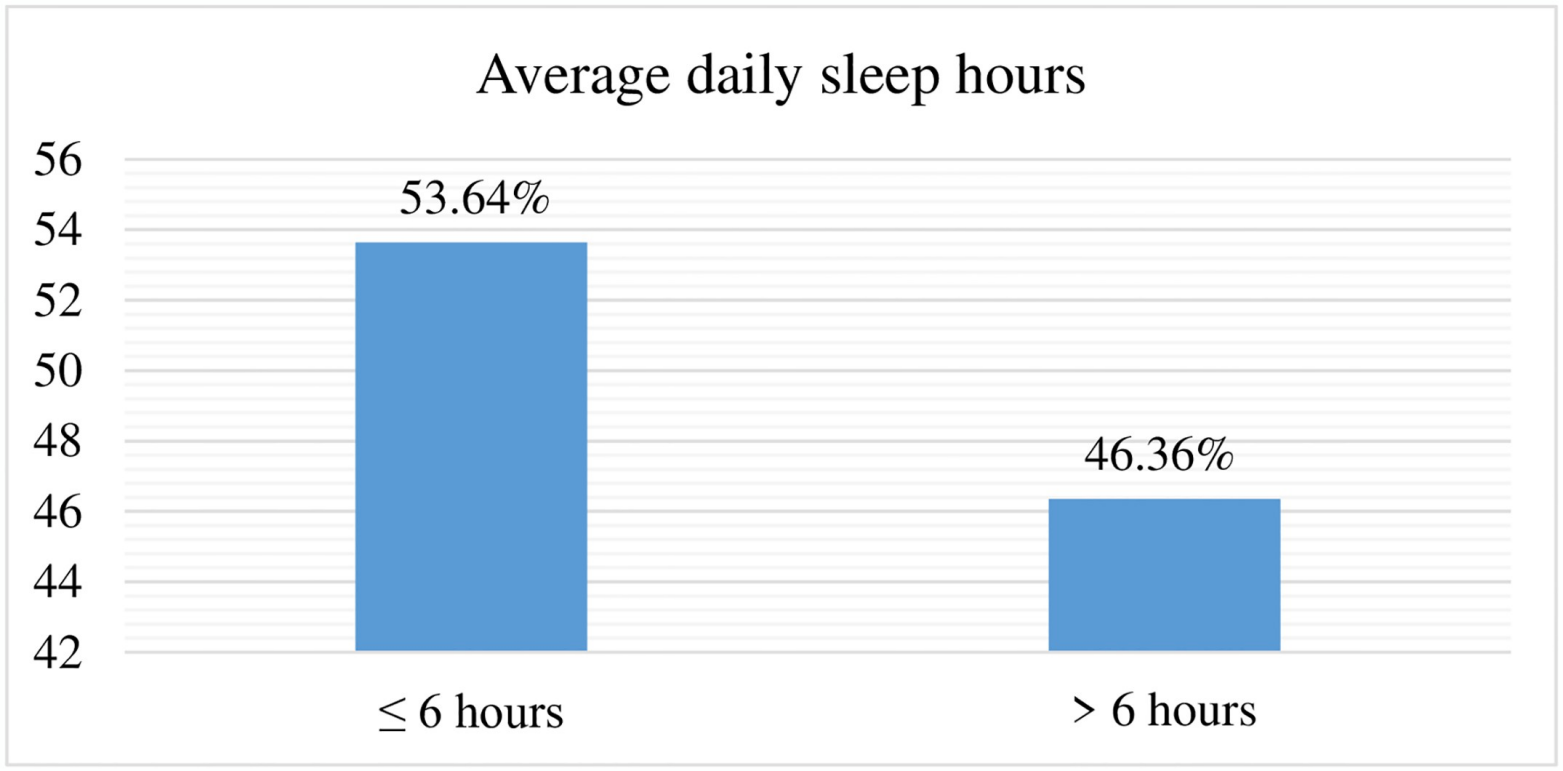
Average daily sleep hours of nurses working in Jimma Zone public hospitals, southwest Ethiopia, 2023 (n = 412).

This study shows that, (53.64%) of the respondents had an average sleep hour of ≤ 6 hours per day (Fig 3).

### Organizational-related factors

#### Healthy work environment

Nurses’ ratings of the healthy work environment (HWE) items were analyzed based on their mean ± SD for overall and each subscale. The overall mean ± SD score for HWE was 2.72 ± 0.71. Based on this overall mean score, 268 (65%) of nurses fell into the category of needing improvement. Considering each subscale, the lowest mean score for HWE was for appropriate staffing, with a mean ± SD of 2.46 ± 0.83. 68.2% of nurses reported that staffing needs improvement. On the other hand, the subclass with the highest mean scores for HWE was authentic leadership, with a mean ± SD of 2.91 ± 0.78. From total respondents 39.4% of nurses reported that leadership was good (Table 4).

**Table 4:** Level of HWE among nurses working in Jimma Zone public hospitals, southwest Ethiopia, 2023 (n = 412).

HWE dimension*	Mean ± SD	Scoring level		
		Needs Improvement	Good	Excellent
		N(%)	N(%)	N(%)
Overall nurse’s HWE rating	2.72 ± 0.71	268(65)	121(29.4)	23(5.6)
Skilled communication	2.82 ± 0.80	248(60.2)	113(27.4)	51(12.4)
True collaboration	2.78 ± 0.76	238(57.8)	140(34)	34(8.3)
Effective decision-making	2.80 ± 0.81	225(54.6)	145(35.2)	42(10.2)
Appropriate staffing	2.46 ± 0.83	281(68.2)	105(25.5)	26(6.3)
Meaningful recognition	2.53 ± 0.88	270(65.5)	110(26.7)	32(7.8)
Authentic leadership	2.91 ± 0.78	203(49.3)	162(39.3)	47(11.4)

* Nurses’ rating of Healthy Work Environment measured on a 5-point Likert scale.

### Level of compassion fatigue

**Fig 4:**
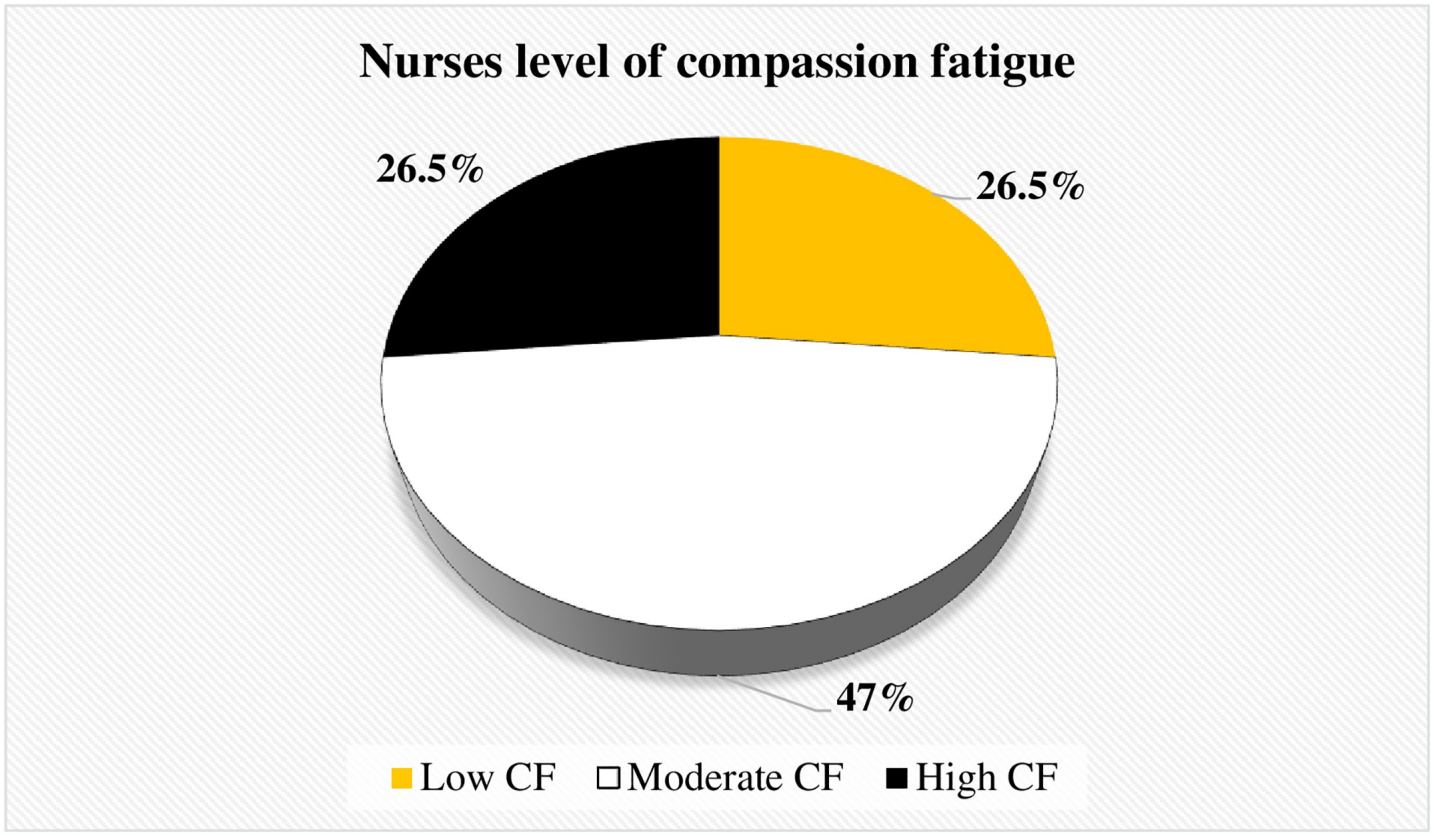
Level of compassion fatigue among nurses working in Jimma Zone public hospitals, southwest Ethiopia, 2023 (n = 412).

This study revealed, of total respondents. 47% of the respondents, had an average level of CF. The minimum and maximum score for CF among nurses was 17 and 49, respectively (Fig 4).

### Factors associated with compassion fatigue

In simple linear regression/bivariate analysis, 14 were identified as candidate variables for multivariable linear analysis based on a p-value < 0.25 significance level. These variables included level of education, marital status, level of hospital, working units, average sleep hours, age, total experience, experience in the current unit, number of children, working hours per week, social support, self-compassion, job-satisfaction, healthy work environment and coping strategy.

In multiple linear regression, after adjusting for other covariates, the multiple linear regression model revealed that six variables were significantly associated with CF at p ≤ 0.05, with a 95% confidence interval. These variables were total experience, working units, Social Support, self-compassion, Support-seeking coping strategy, and average sleep hours. The model also showed that 69% of the variance in the compassion fatigue score among nurses was explained by the combined effects of those variables, and the model was significant (Adjusted R^2^ = 0.69, p = <0.001).

Accordingly, after adjusting for other covariates, the total experience was significantly associated with CF [β = -0.04; 95%CI (-0.06, -0.01); p = 0.005]. This suggests that as the work experience increases by one year, the level of CF decreases by 0.04. Furthermore, the study revealed that controlling for other covariates, perceived social support was negatively and significantly associated with compassion fatigue [β = -0.13; 95% CI (-0.17, -0.08); p<0.001]. This means that as perceived social support increases by one unit, compassion fatigue decreases by 0.13.

Similarly, after controlling for other variables, self-compassion was negatively and significantly associated with CF [β = -0.09; 95% CI (-0.14, -0.03); p = 0.003]. This suggests that as self-compassion increases by one unit, CF decreases by 0.09. Additionally, Support seeking (coping strategy) was also negatively and significantly associated with CF [β = -0.23; 95% CI (-0.42, -0.04 p = 0.017]. This implies that as support-seeking increases by one unit, CF decreases by 0.23.

Working unit was also found to be significantly associated with CF. Specifically, the level of CF among nurses working in the emergency ward was 0.36 higher than nurses working in the medical ward [β = 0.36; 95% CI (0.2, 0.51); p <0.001]. Similarly, the level of CF among nurses working in the ICU was 0.38 higher than nurses working in the medical ward [β = 0.38; 95% CI (0.21, 0.54); p<0.001]. Also, the level of CF among nurses working in the pediatric ward was 0.23 higher than nurses working in the medical ward [β = 0.23; 95% CI (0.10, 0.36); p < 0.001].

Additionally, the study found that average sleep hours per day were negatively associated with CF. The level of CF among nurses who slept for six hours or less was 0.46 less than nurses who slept for more than six hours [β = 0.46; 95% CI (0.35, 0.57); p<0.001]. This suggests that nurses who slept for six hours or less were at a higher risk of experiencing compassion fatigue than nurses who slept for more than six hours (Table 5).

**Table 5:** Multiple linear regression of compassion fatigue and associated factors among nurses working in Jimma Zone public hospitals, southwest Ethiopia (n = 412).

Variables		Crude unstandardized regression coefficient (β)	Adjusted unstandardized regression coefficient (β) (95% CI)	P-value	Model summary
Total Experience		-0.09(0.11, -0.08)	-0.04 (-0.06, -0.01)	0.004	Adjusted R^2^ = 0.69 P = <0.001
Perceived social support		-0.25 (-0.10, -0.21)	-0.13(-0.17, -0.08)	<0.001	
Self-compassion		-0.42 (-0.48, -0.35)	-0.09 (-0.14, -0.03)	0.003	
Coping strategy	Support seeking	-0.69 (-0.82, -0.56)	-0.23(-0.42, -0.04)	0.017	
Average daily sleep hours	> 6 hours	0	0	<0.001	
	≤ 6 hours	1.00 (0.89, 1.11)	0.46(0.35, 0.57)		
Working units	Medical ward	0	0		
	Emergency ward	0.84 (0.58, 1.10)	0.36(0.21, 0.51)	<0.001	
	ICU	0.93 (0.64, 0.21)	0.38 (0.21, 0.54)	<0.001	
	Pediatric	0.78 (0.51, 1.06)	0.23 (0.10, 0.36)	<0.001	

0 = Reference Group.

## Discussion

The study revealed that 26.50% (95% CI (22.18%, 30.73%)) of the respondents had high levels of CF, indicating that more than one in four nurses reported experiencing a high level of CF. Although no study was identified in Ethiopia to enable a direct comparison, the findings of this study is consistent with the literature from other countries. The prevalence of high levels of CF in the present study was consistent with studies conducted in New Zealand, South Africa, and Greece, in which 22.9%, 27.9%, and 22.9% of nurses experienced a high level of CF, respectively [35, 41, 42].

The result of this study was higher compared to other earlier studies conducted in various countries. Studies from Jordan, Australia, and the US reported that none of the respondents had a high level of CF. Also, studies from China and Egypt revealed that 0.2% and 2.9% of the respondents had high levels of CF, respectively [21, 31, 43–45]. This inconsistency in the prevalence of high levels of CF might be attributed to several factors. One contributing factor might be the convenience sampling technique and the small sample size used in previous studies from Jordan, Australia, and the US. Furthermore, this inconsistency could be explained by differences in data collection methods, such as email surveys, which led to a lower response rate, 38% in Australia and 44% in America [31, 43].

Another contributing factor might be the difference in average weekly working hours. For instance, nurses in America (35 hours) and Egypt (38 hours) had lower average weekly working hours, which resulted in fewer exposure times to suffering patients and, thus, less likely to develop CF. The difference in the inclusion criteria could also be another contributing factor, as the Jordanian study included nurses with more than two years of experience. In addition, variations in the length of average work experience, regions, resources, and healthcare system infrastructure could have contributed to the differences observed in the prevalence of high levels of CF. The lack of psychological support for nurses working in developing countries like Ethiopia could also contribute to the difference [46]. Additionally, previous studies indicated that nurses were emotionally prepared through specialty education in their working units, well supported by managers, and had effective organizational processes to enable functioning despite the trauma and suffering they saw in their environment [31, 43].

However, the prevalence of high levels of CF in this study was lower than in other studies conducted in Greece, Portugal, Uganda, and Poland, in which 73.9%, 58.6%, 49.11%, and 43.6% of the respondents had high levels of CF, respectively [25, 26, 47, 48]. This difference might be attributed to the working unit and study period variations. This difference might be explained by previous studies in Portugal, Poland, and Uganda conducted in units considered high risk for CF, like in emergency and intensive care units. Similarly, a study in Greece was also done in labor wards, in which a significant portion of nurses witnessed a traumatic birth event, cared for a woman after stillbirth, and cared for a woman with intense depression or stress, which impacted nurses’ psychological well-being to the extent of experiencing CF [47]. Additionally, previous studies were conducted during peak times of the COVID-19 pandemic, which affects nurses’ personal and professional lives and interpersonal relationships of nurses, increasing their risk of developing CF. Nurses working during the COVID-19 pandemic are under great physical-mental stress and are exposed to many traumatized patients in a short time, making them more prone to CF [26, 45]. Moreover, their educational background could also be a contributing factor. In a study carried out in Uganda, most participants had diplomas or less.

The study finding shows a statistically significant negative association between total working experience and CF. These results align with previous studies conducted in various settings, including Uganda, the United States, China and Southern California [26, 49–51], which reported that nurses who have been in the profession for longer tend to have a lower CF. This consistency could be attributed to several factors, such as increased professional skills and knowledge, improved self-care practices, and enhanced emotional regulation over time with their experience [52].

The study finding also indicates that nurses working in emergency, ICU, and pediatric units were significantly associated with increased CF compared to those working in medical wards. This finding is congruent with previous two research done in Spain and Greece [24, 53, 54]. This similarity could be explained as working in the emergency ward and ICU exposing nurses to stressful environments, frequent mortality, and challenging daily work routines [11]. Furthermore, nurses’ sensitivity to painful procedures, serious injuries, or death in children, especially if their patient is the same age as their child, could be triggers of CF among nurses working in the pediatrics unit [55].

The study’s results have also shown that perceived social support had a significant negative association with CF. This finding coincides with previous research conducted in china and Poland [22]. This concurrence might be because social support is a psychosocial resource that makes nurses feel comfortable, respected, and happy as they feel cared for, loved, valued, and needed by others. Another explanation is that social support helps nurses concentrate better on their work, become more confident in solving work-related problems, and reduce negative emotions, which could decrease CF [21].

In addition, the study has revealed that self-compassion was found to have a negative association with CF, this implies that as self-compassion increases, CF decreases. This finding is consistent with a study conducted in the UK, which showed a significant negative relationship between self-compassion and CF [56]. A possible explanation might be that self-compassion can help prevent CF by reducing negative thoughts and emotions, leading to better psychological functioning and greater life satisfaction [57]. In addition, self-compassion allows nurses to hold their suffering with a sense of warmth, connection, and concern, which can help reduce the symptoms of CF [58]. Furthermore, self-compassion helps improve resilience, enabling nurses to thrive in the face of difficulties and can help increase their coping capacity [59].

Similarly, support-seeking of the coping strategy was negatively associated with CF. The finding of this study is consistent with the study conducted in Egypt, Jordan, and Iran [21, 44, 60]. A possible justification might be that seeking social support within the work environment helps to enhance nurses’ ability to accommodate the stressful nature of working as a nurse. Furthermore, seeking support offers emotional support such as trust and respect, and these effects can play an essential role in reducing the symptoms of CF.

Lastly, the study discovered that nurses who slept for six hours or less had a higher level of CF than those who slept for more than six hours. This result is consistent with two studies conducted in China [39, 61]. One possible explanation is that insufficient sleep reduces the number of sleep cycles, worsening the symptoms of CF. Sleep is also a biological necessity that helps maintain homeostasis, enhances mental health, and reduces CF [61].

This study had several strengths, including that the study contained primary, adequate, and representative samples from a multicenter selected randomly. Therefore, the findings might be extrapolated to nurses working in Jimma Zone public hospitals and beyond. However, the study also had some limitations. The cross-sectional design used in the study does not allow inference of causality from the observed relationship, and it did not assess changes in the CF of participants over time. Furthermore, the self-reported nature of the questionnaires may have caused an over or underestimation of results. Additionally, the proportion of sample distribution on some variables, including educational and marital status was uneven. Finally, literature is scarce on this topic in our country, so comparing study results with other countries where the health institutions setup, health policy, and other factors are quite different.

### Conclusion

The study revealed that more than one in four nurses reported having a high level of CF. This finding indicates that many nurses were affected by negative experiences of caring for patients, which may adversely impact nurses’ well-being, patients, and healthcare outcomes. This alarming phenomenon warrants the need for developing strategies to mitigate CF among nurses, especially those working in emergency, pediatrics, and intensive care units. This study also showed that variables including working experience, perceived social support, self-compassion, and support-seeking coping strategies were negatively associated with CF. However, working in emergency, ICU, and pediatrics units and sleeping less than or equal to six hours per day were positively associated with CF.

## Supporting information

S1 Data (SAV)
